# Therapeutic Advances Against ZIKV: A Quick Response, a Long Way to Go

**DOI:** 10.3390/ph12030127

**Published:** 2019-08-30

**Authors:** Juan-Carlos Saiz

**Affiliations:** Department of Biotechnology, Instituto Nacional de Investigación y Tecnología Agraria y Alimentaria (INIA), 28040 Madrid, Spain; jcsaiz@inia.es; Tel.: +34-91-347-8770

**Keywords:** Zika virus, vaccines, direct acting antivirals (DAAs), host acting antivirals (HAAs)

## Abstract

Zika virus (ZIKV) is a mosquito-borne flavivirus that spread throughout the American continent in 2015 causing considerable worldwide social and health alarm due to its association with ocular lesions and microcephaly in newborns, and Guillain–Barré syndrome (GBS) cases in adults. Nowadays, no licensed vaccines or antivirals are available against ZIKV, and thus, in this very short time, the scientific community has conducted enormous efforts to develop vaccines and antivirals. So that, different platforms (purified inactivated and live attenuated viruses, DNA and RNA nucleic acid based candidates, virus-like particles, subunit elements, and recombinant viruses) have been evaluated as vaccine candidates. Overall, these vaccines have shown the induction of vigorous humoral and cellular responses, the decrease of viremia and viral RNA levels in natural target organs, the prevention of vertical and sexual transmission, as well as that of ZIKV-associated malformations, and the protection of experimental animal models. Some of these vaccine candidates have already been assayed in clinical trials. Likewise, the search for antivirals have also been the focus of recent investigations, with dozens of compounds tested in cell culture and a few in animal models. Both direct acting antivirals (DAAs), directed to viral structural proteins and enzymes, and host acting antivirals (HAAs), directed to cellular factors affecting all steps of the viral life cycle (binding, entry, fusion, transcription, translation, replication, maturation, and egress), have been evaluated. It is expected that this huge collaborative effort will produce affordable and effective therapeutic and prophylactic tools to combat ZIKV and other related still unknown or nowadays neglected flaviviruses. Here, a comprehensive overview of the advances made in the development of therapeutic measures against ZIKV and the questions that still have to be faced are summarized.

## 1. Introduction

Human and animal health has to face changes in the ecology of pathogens resulting from climate warming, vector colonization of new geographical niches, global trade, and human behavior. In this sense, emerging viruses represent a worrisome challenge because of their current worldwide spread, as exemplified by the outbreaks of the West Nile virus (WNV) in North America in 1999 [1], Chikungunya virus (CHIKV) in Asia in 2004–2005 [2], or Ebola virus (EBOV) in Western Africa in 2014 [3]. However, the more recent threatening has been caused by the emergence and dissemination of Zika virus (ZIKV) in the Americas in 2015 [4]. The virus has provoked more than 580,000 autochthonous suspected disease cases in the Americas since then [5], causing social and medical alarm mainly due to the evidence of a causal link between ZIKV and several congenital injuries, like microcephaly and ocular lesions, as well as to its association with neurological disorders, such as Guillain–Barré syndrome in adults [6]. All these events led the World Health Organization (WHO) to the declaration of a Public Health Emergency of International Concern (PHEIC) in February 2016 [7]. Since then, and even though the number of reported clinical cases has decreased a lot [8], a great effort has been conducted by the scientific community to understand the virus biology and pathogenesis, to improve diagnostic tools, and to develop therapeutic and prophylactic alternatives.

ZIKV is a mosquito-borne flavivirus (family *Flaviviridae*), which genome (about 11 kb in length) encodes a single open reading frame translated into a viral polyprotein that, after cotranslational and postranslational proteolytic cleavage, generates 10 mature proteins: Three structural proteins [C (capsid), prM/M (membrane) and E (envelope)] and seven non-structural proteins (NS1, NS2A, NS2B, NS3, NS4A, NS4B and NS5) [4,9].

The natural course of the ZIKV infection is usually asymptomatic or produces a relatively mild illness with a wide range of symptoms that go from fever, rush, and headache, to joint and muscle pain, and that usually ends up in an uneventful recovery [6,10]. Nevertheless, since ZIKV colonized the Americas, it has shown an increased virulence and rapid spread, and it has been associated with an unexpected rise of microcephaly cases in fetuses and newborns, severe ocular lesions, and a remarkable increase in Guillain–Barré syndrome (GBS) cases [10,11]. Additionally, ZIKV can also be sexually transmitted and persist in the male genital tract, which may lead to infertility [12].

Albeit a huge amount of research has been conducted in the last three years, which extensively surpasses that of any other recently emerging pathogen [13], there is still a lack of effective ZIKV vaccines or antivirals. Our current knowledge about ZIKV molecular mechanisms of pathogenesis, including the development of amenable animal models [14,15,16] and the elucidation of the crystal structure of some of the ZIKV proteins (C, NS1, NS2b-NS3, NS3, and NS5) [17], are certainly contributing to develop vaccine prototypes and to identify antiviral candidates; however, we are still far from controlling the virus. Here, a comprehensive overview of the advances made in the development of therapeutic measures against ZIKV is given.

## 2. Vaccines

Nowadays, measures such as vector control and surveillance programs, and avoiding mosquito bites are the more effective ones against the ZIKV infection. In addition, as pregnant and fertile women are the more at risk population, authorities from some countries recommend to abstain from unprotected sex if they, or their partners, live or travel to endemic regions. Therefore, development of efficient vaccines is more probably the best way to fight the ZIKV disease. 

Several questions should be posed before a vaccine can be licensed to use in the clinical practice. In addition to its effectiveness, safety for pregnant women is mandatory [18], as they will be the main target for the ZIKV vaccine administration and other flavivirus vaccines, such as those for the Japanese encephalitis virus (JEV) or yellow fever virus (YFV), are usually contraindicated for them. Similarly, because different flaviviruses co-circulate in the same geographical areas, the possibility that a vaccine could generate cross-reactive antibodies and, thus, induce antibody dependent enhancement (ADE), should be taken into account. Additionally, as the ZIKV vaccine will be used preferably in developing countries, cost is another important point to take into account. In this sense, the WHO has published an updated target product profile (TPP) for a Zika vaccine that could be used in an outbreak response scenario [19].

So far, in less than three years, a large number of candidate vaccines has been evaluated in the early stages, and some of them have even entered into clinical trials (Table 1). Almost all available approaches have been rehearsed: Purified inactivated and live attenuated viruses, nucleic acid (DNA or RNA) based candidates, virus-like particles, subunit elements, and recombinant viruses.

### 2.1. Purified Inactivated Vaccines

Several purified inactivated ZIKV vaccines have been tested in mice and in non-human primates (NHP). Overall, the production of high levels of neutralizing antibodies, reduction of viremia, and protection of interferon deficient mice challenged with homologous and heterologous viral strains, and passive protection after administration of immunoglobulins from vaccinated animals, which seems to correlate with neutralizing antibody titers, have been reported [20,32]. One of these vaccines [20] entered the phase I clinical trial in India (CTRI/2017/05/008539) [21]. Another purified formalin-inactivated Zika virus vaccine (ZPIV) tested in NHP also induced specific neutralizing antibodies and full protection after viral challenge [22]. This ZPIV vaccine has entered too in phase I, placebo-controlled, double-blind trials (NCT02963909, NCT02952833, NCT02937233, NCT03343626, and NCT03425149). Initial reports indicate that most of the enrolled participants seroconverted, developed antibody titers higher than the threshold recorded in animal studies, and that the passive transfer of purified neutralizing IgG from vaccinated participants protected mice against the ZIKV infection [23].

### 2.2. Live Attenuated Vaccines

Different approaches have been tested as live attenuated vaccine candidates. A prototype engineered by deleting 10 nucleotides from the 3’ untranslated region of the viral genome (10-del ZIKV) induced high neutralizing antibody titers and a strong cellular response, completely reduced viremia in interferon-receptor deficient mice, and protected CD1 newborn mice from intracranial inoculation of a lethal strain [33]. Even more, the same group demonstrated that a single dose of this vaccine diminishes levels of viral RNA in maternal, placental, and fetal tissues in challenged mice, preventing viral transmission during pregnancy, as well as testes damage and oligospermia in vaccinated male mice [33]. This vaccine prototype also protected NHP from ZIKV infection, but animals showed an anamnestic antibody response when challenged, indicating that the virus did replicate and, thus, there were no sterilizing immunity [34]. Immunization with an attenuated vaccine due to a deletion of nine amino acids in the viral capsid protein also elicited protective immunity that completely prevented viremia, conferred protection after challenge, and avoided virus transmission in immunodeficient mice [35]. Another attenuated prototype encoding a non-glycosylated NS1 protein diminished viral RNA levels in maternal, placental, and fetal tissues, and protected against placental damage and fetal demise when tested in dams early after pregnancy [36].

### 2.3. Nucleic Acid (DNA or RNA) Vaccines

Several DNA-based vaccines expressing the viral prM/E proteins have been evaluated in animal models, including humanized mice. Overall, these candidates induce the production of specific humoral and cellular responses, reduce viremia, protect against viral challenge, and confer protection after passive transfer of immune serum from both vaccinated mice and NHP, and, in some cases, prevent vertical virus transmission [24,27,37,38,39]. A DNA (prM/E) prototype has been shown to protect mice against ZIKV-associated damage to the testes and sperm after viral challenge, preventing viral persistence in the testicles [39]. These types of vaccines have also been effective in protecting NHP against the ZIKV challenge [32]. Even more, one of the above mentioned DNA vaccine (GLS-5700), which had been previously tested in mice [25], has entered clinical trials phase I (NCT02809443 and NCT02887482) [26]. In these studies over 80% of the vaccinated individuals elicited neutralizing antibodies, and inoculation in immunodeficient mice of their post-vaccination sera protected most of the animals against a lethal viral challenge. Likewise, and based on previous data obtained after mice vaccination [27], preliminary results of two other trials (NCT02840487 and NCT02996461) have been reported, one evaluating a chimeric ZIKV-JEV plasmid and the other a ZIKV wild-type plasmid. Both candidates were safe and well tolerated, and strong specific antibody and cellular responses were achieved with the chimeric one [28]. The one encoding the wild type ZIKV has advanced to the phase II clinical trial (NCT03110770).

Similar approaches have been conducted using RNA-based vaccines encoding also the prM/E region of the viral genome and administered as nanoparticles (NP). An early report showed that a candidate tested in immunocompetent mice induced the production of specific antibodies and a CD8 + response against a unique H-2Db-restricted epitope was identified [40]. Another non-replicating lipid-nanoparticle-encapsulated nucleoside-modified mRNA (mRNA-LNP), encoding the prM/E and expressed in cells to produce virus-like particles (VLPs), induced a potent, sterilizing, and durable neutralizing antibody response in mice and NHP, and protected both of them against the ZIKV infection [29]. Similarly, another mRNA-LNP candidate also protected against the ZIKV infection and conferred sterilizing immunity in mice [36]. Still more, taking into account the possibility that the vaccine could induce cross-reactive antibodies against DENV and, thus, induce antibody dependent enhancement (ADE), the authors introduced mutations in the conserved fusion-loop epitope of the E protein [36]. These mutant vaccines not only protected against the ZIKV challenge, but also diminished the production of antibodies that may enhance the DENV infection in cells and mice [25]. Even more, the same RNA-LNP construct (mRNA-1325) diminished levels of viral RNA in maternal, placental, and fetal tissues [30], and has advanced to clinical trials (NCT03014089).

### 2.4. Virus-like Particles Vaccines

In addition to the above mentioned modified lipid-encapsulated RNA vaccines encoding the prM/E region that render virus-like particles (VLPs), several other groups have described the immunogenicity of this kind of prototypes. VLPs assembled by co-expressing the C/prM/E and the NS2B/NS3 viral proteins stimulated the production of high virus neutralizing antibodies in mice [41,42]. Likewise, VLPs produced by the expression of just the prM/E proteins in HEK293 cells induced neutralizing antibodies that were protective in infected mice and in those receiving passive transfer of purified IgG [43]. A different approach was conducted by using recombinant VLPs based on the hepatitis B core antigen (HBcAg) that incorporates the ZIKV E protein domain III (DIII) and were produced in *Nicotiana benthamiana* plants. This candidate elicited potent humoral and cellular responses in mice and did not enhance the infection of DENV in Fc gamma receptor-expressing cells [44].

### 2.5. Subunit Vaccines

Purified soluble ZIKV E protein, expressed in *Drosophila melanogaster* cells or *E. coli*, induced virus-specific neutralizing antibodies, and avoided viremia when inoculated in immunocompetent mice [45,46]. When assayed in NHP, one prototype produced in insect cells elicited high neutralizing antibody titers, conferred sterilizing immunity, and protected mice after passive transfer of plasma from vaccinated NHPs [47]. Likewise, an N-terminal truncated E (∆E) protein, expressed in either of the two systems, elicited ZIKV-specific humoral and cellular response in immunocompetent mice, and passive transfer of sera from immunized animals conferred full protection against the lethal ZIKV challenge in newborns mice [48,49]. A similar ∆E protein has also been shown to reduce ZIKV-infected cells in the brains of fetal or suckling mice, preventing the onset of microcephaly [50].

Purified ZIKV envelope domain III (DIII) has also been expressed in *D. melanogaster* cells, *E. coli*, and *Nicotiana benthamiana* plants. These constructs induced humoral and cellular responses when administered to mice, and passive transfer of sera protected recipient mice against the lethal ZIKV [49,51,52]. In addition, they did not enhance the infection of DENV when tested in Fc gamma receptor (FcγR)-expressing cells [51,52]. Likewise, a fragment (aa 298-409) of the DIII expressed in 293T cells stimulated long-term immunogenicity and protected immunodeficient mice and newborns from vaccinated mothers against the virus challenge, and also pups from naïve mothers that received a passive transfer of specific antibodies [53].

### 2.6. Recombinant Vaccines

Different viruses have been assayed as a backbone for the development of ZIKV recombinant vaccines. Adenovirus strains of various serotypes have been engineered to include the ZIKV structural proteins. A recombinant chimpanzee adenovirus type 7 (AdC7) expressing ZIKV M/E proteins elicited durable humoral and cellular responses, and protective and sterilizing immunity against heterologous ZIKV infection in mice, and also protected against testes damage [54]. Several human adenoviruses (Ad4, Ad5 and Ad26) encoding either the E alone or the prM/E have shown to induce a durable antibody and cellular responses in mice and NHP, reducing viral titers in tissues and blood, and protecting mice from viral challenge [55,56,57].

Similar approaches have been conducted by using modified vaccinia Ankara (MVA) as a vector. One prototype encoding the ZIKV prM/E elicited neutralizing antibodies, induced a potent and polyfunctional ZIKV-specific CD8 + T cell response, and significantly reduced viremia in mice [58]. Another MVA candidate expressing the ZIKV-NS1 also provided robust humoral and cellular responses, and afforded 100% protection in mice against a lethal intracerebral challenge [59]. This line of attack is interesting because it could circumvent potential ADE, as it does not encode the E protein. By using the recently developed vaccinia-based Sementis Copenhagen Vector (SCV), a multi-pathogen vaccine encoding the structural proteins of chikungunya and Zika viruses was shown to induce neutralizing antibodies against both viruses and to reduce viremia in mice [60]. In addition, it conferred protection against chikungunya-related arthritis, and ZIKV fetal/placental and testis infection.

As in the case of adeno and vaccinia viruses-based vaccines, vesicular stomatitis virus (VSV) has also been evaluated as a platform for ZIKV vaccines. Immunization with a VSV construct expressing the GP of the EBOV and the ZIKV prM/E, or a prM/ΔE, resulted in complete protection of challenged mice [61]. Likewise, when the ZIKV prM-E-NS1 was produced as a VSV recombinant construct, it induced the ZIKV specific humoral and cellular responses that protected against the ZIKV challenge [62]. Furthermore, a VSV-based CHIKV (E3-E2-6K-E1) and ZIKV (M/E) bivalent vaccine induced neutralizing antibody responses to both CHIKV and ZIKV in mice and protected them against infection with either viruses [63].

Recently, results obtained after immunization of mice with a live-attenuated measles virus (MV) strain encoding the ZIKV prM/E have been reported [31]. Specific neutralizing antibodies and cellular responses, reduction of viral titers in sera and organs, and prevention of infection of the fetus were observed in immunized animals. This candidate has entered the phase I clinical trial (NCT02996890).

Finally, advantages have been taken of the already available vaccines against other related flaviviruses. So that, a recombinant chimeric ZIKV vaccine candidate that expresses the ZIKV prM/E proteins using the licensed JEV live-attenuated vaccine (SA14-14-2) as a backbone elicited robust and long-lasting immune responses and conferred complete protection against the ZIKV challenge both in mice and NHP [64]. Even more, the vaccine also protected against the ZIKV placental and fetal damage during pregnancy [64]. Likewise, when the prM/E proteins of the live attenuated yellow fever 17D vaccine virus (YF 17D) were replaced by those of ZIKV, it was shown a significantly induction of neutralizing antibodies and a reduction of viral loads in organs, avoiding neuroinvasion, and protecting against the ZIKV challenge in mice [65]. A ZIKV (prM/E)-YF 17D variant with adaptive mutations in the E protein also drove to seroconversion and to a strong cellular response in vaccinated mice that conferred full protection against homologous and heterologous viral challenge, and protected fetus from intra-placental challenged vaccinated dams [66]. Improvements of this kind of prototypes by modifying the cleavage site between the pM and M, which are compensated by mutations in the E protein, induced high neutralizing antibody titers in mice and protected against the heterologous viral challenge [67].

### 2.7. Therapeutic Vaccination

As mentioned above, the success of passive transfer of sera from vaccinated mice, NHP, and humans in protecting mice against the ZIKV infection has been extensively reported. In this regard, an early study demonstrated that an EDIII-specific antibody protected mice from lethal ZIKV infection [68]. A DENV-specific antibody against an E-epitope has also been shown to cross-neutralize ZIKV and protect against the ZIKV infection when administered to NHP [69]. Later on, it was reported that neutralizing the convalescent serum from a donor who recovered from the ZIKV infection prevented virus replication and ZIKV-induced microcephaly when inoculated in pregnant mice [70]. Therapeutic human monoclonal antibodies (mAbs) have also been evaluated in mice. So that, a subset of mAbs from patients recovered after the ZIKV infection were shown to neutralize homologous and heterologous viral strains, and administration of one of them in mice reduced tissue pathology, and placental and fetal infection [71]. Similarly, a single injection of ZIKV-specific human neutralizing mAbs induced different levels of protection against the ZIKV infection in adult mice and in utero [72]. A cocktail of three ZIKV-neutralizing mAbs from a ZIKV-infected patient, which were engineered to the abrogate Fcγ receptor binding to eliminate a potential ADE effect, completely prevented viremia when administered to NHP one day before the ZIKV challenge [73]. Likewise, another report showed that four non-neutralizing mAbs from an infected patient that target the non-structural protein NS1 can engage FcγR without inducing ADE in vitro, and that one of them was protective against lethal homologous and heterologous challenges in mice, an effect that was Fc-dependent [74].

### 2.8. Future Perspectives for ZIKV Vaccines.

As described above, in as little as three years a great number of vaccine candidates has been tested in animal models using many different approaches that, overall, have led to hopeful results. Induction of strong specific humoral and cellular responses, decrease or undetectability of viremia and viral RNA in organs (including placenta, foetal tissues, and testes), prevention of viral transmission during pregnancy and of the onset of microcephaly, and protection in experimental animal models, which seems to correlate with the development of high titers of neutralizing antibodies, have been achieved. Additionally, experiments with passive transfer of immune sera from vaccinated individuals and human mAbs point to therapeutic vaccination as a possible option for the ZIKV control. In fact, several candidates have already entered in clinical trials (Table 1) [75].

In any case, and even though much progress has been accomplished, there is still a long way to go before a prophylactic vaccine can be licensed for use in clinical practice. So that, several considerations should be kept in mind. Although there are evidences pointing that neutralizing antibodies are likely a surrogate for protection, many preclinical tested vaccines did not induce sterilizing immunity and, thus, this point remains to be clarified. In fact, it seems that, in comparison with other flaviviruses, higher levels of neutralizing antibodies are required to protect animals, and probably humans, against the ZIKV infection. Likewise, the durability of the induced immunity has still to be more deeply evaluated. On the other hand, as ZIKV circulates in regions endemic for other related flaviviruses (DENV, YFV, WNV, etc.) another important point is to assure that vaccine implementation would not induce undesirable results like the enhancement of a secondary infection due, for instance, to ADE. In addition, ideally, a vaccine must also prevent infection of reproductive tissues to avoid sexual transmission. Even more, as the main target population that will benefit ZIKV vaccines are fertile and pregnant women and, since other licensed flavivirus vaccines (JEV or YFV) are usually contraindicated for them, safety should be fully evaluated in this population. Moreover, and albeit positive results have been obtained in mice, this is not an adequate model for women pregnancy, and therefore, this point must be carefully taken into account before vaccine campaigns implementation. On top of that, since the number of new ZIKV cases recorded has drastically dropped and other related flaviviruses co-circulate in the same geographical regions, further clinical endpoint trials efficacy will be difficult to undertake. In any case, ZIKV is still circulating at low levels in some areas and its re-emergence in naïve populations or in areas where population immunity wanes remains a real possibility; therefore, the public health need for a ZIKV vaccine persists.

## 3. Antivirals

Nowadays there is no licensed specific antiviral against ZIKV [76], or any other flavivirus [77], and treatments are palliative and directed to symptoms relief. Hence, as in the case of vaccines development, in a short time many efforts have been conducted to assay dozens of possible antiviral candidates by using many different approaches and tools (in vitro and *in silico* assays, diverse viral and cell lines, including primary and pluripotent stem-cells, infectious clones, and replicons) [15]. Two major lines of investigation are being evaluated: Search for direct acting antivirals (DAAs) based on interference with viral components, and search for host-acting antivirals (HAAs) based on the inhibition of host factors co-opted for the virus to complete its infectious cycle. In both approaches two important points that ZIKV antivirals have to face is their safety for pregnant women and their ability to cross the blood-brain barrier (BBB) to combat the infection in nervous tissues [78]. With this in mind, efforts have been done by testing of natural compounds, screening of libraries from different sources, and repurposing of drugs with the known antiviral activity that have allowed the identification of several antiviral candidates directed to viral structural proteins and enzymes and to cellular factors.

### 3.1. Direct Acting Antivirals (DAAs)

DAAs (Table 2) are those compounds that directly interfere with viral components, either structural proteins or enzymes, for which many of their crystal structures (C, NS1, NS2b-NS3, NS3, and NS5) have been resolved [17], what is undoubtedly helping to find more precise antivirals. 

#### 3.1.1. Compounds Acting Before Viral Binding

Administration of convalescent sera and neutralizing antibodies suppress ZIKV multiplication and inhibits cell death in infected fetal brains, preventing microcephaly in mice and protecting NHP [68,69,70,71,72,73,74,79]. In this line, as commented above regarding vaccines development, many authors have reported that the passive transfer of immune sera from vaccinated individuals protects mice against the ZIKV challenge, thus pointing to the use of therapeutic antibodies to combat the ZIKV infection. However, as in the case of vaccines implementation, the possibility of undesirable side effects due to cross-reactive antibodies against other flaviviruses, such as ADE in the case of dengue infections, need to be carefully contemplated.

Different compounds impair viral fusion to the cellular membrane, mostly by directly binding to the viral envelope E protein and preventing E-mediated membrane fusion. So that, the synthetic peptide Z2, which is able to penetrate in fetal tissues, avoids vertical transmission and protects against the ZIKV challenge in mice [80]. Porphyrins like Co-protoporphyrin IX (CoPPIX) and Sn-protoporphyrin IX (SnPPIX) induce viral envelope protein loss, affecting viral morphology and entry into target cells [81]. Benzoxazine monomer derived carbon dots (BZM-CDs) [82], polyoxometalates (POMs) [83], cyanohydrazones [84], synthetic carbohydrate receptors (SCRs) [85], and some other small molecules [86,87,88] also inhibit ZIKV infection in cell culture, and reduce viral replication in mice [89]. 

Compounds exerting a viricidal activity, such as crotoxin, from the *Crotalus durissus terrificus* venom [90], *Aphloia theiformis* and *Psiloxylon mauritianum* extracts [91,92], and polyphenols like epigallocatechin gallate or delphinidin, present in many natural products, also exhibit anti-ZIKV activity [93,94,95], as does the natural flavonoid pinocembrin that impairs the ZIKV infection in culture cells, although it seems to act on post-entry processes [96].

#### 3.1.2. Antivirals Targeting the ZIKV NS5 Polymerase and Methyltransferase Domains

The NS5 protein is the RNA dependent RNA polymerase (RdRp) responsible of viral genome replication. Nucleoside analogs/derivatives target viral but not cellular polymerases and, after their incorporation into the viral nascent RNA chain, abrogate viral RNA replication. These compounds are usually safe for use in humans 3 [143] and, thus, they have been widely evaluated as ZIKV antivirals in cell culture and, in a few cases, in animal models.

Several inhibitors of the inosine monophosphate dehydrogenase (IMPDH), such as ribavirin, a well-known antiviral against many RNA viruses [144], and favipiravir (or T-705) inhibits ZIKV multiplication in different cell lines, including human ones [97,98,99,100,101,102], and abrogate viremia in ZIKV-infected susceptible mice [99], as do merimepodib [103], and mycophenolic acid [104,105,106,107], although the later showed significant cell toxicit [104]. Azathioprine, another purine synthesis inhibitor and immunosuppressant, impairs ZIKV replication in cell culture [108], but it is not recommended for use during pregnancy. Methotrexate (MTX), an anti-cancer chemotherapy and anti-rheumatoid agent, exerts its antiviral activity by inhibiting the dihydrofolate reductase (DHFR) in different cell lines, restricting the synthesis of adenosine triphosphate and disrupting ZIKV replication [109]. Atovaquone, a FDA pregnancy category C drug used to prevent parasitic infections, impairs ZIKV virus production in human cells by inhibiting pyrimidine biosynthesis, and limits ZIKV infection in an ex vivo human placental tissue model [110]. Likewise, 6-methylmercaptopurine riboside [111], 2′-C-methylguanidine, 2′-C-methyladenosine, and 7-deaza-2′-C-methyladenosine inhibit ZIKV replication in cell cultures [105,112,113], and the later one reduces viremia and retards disease signs in mice [113]. Finally, NITD008 [114] and BCX4430 [115] protects immunosuppressed mice, although the later one does not reduce viral loads. 

ZIKV replication in cell culture is also affected by a variety of inhibitors of pyrimidine biosynthesis, such as brequinar and CID 91632869 [108], which effect may be due not to pyrimidine privation but to cellular immune response induction [116,145]. Gemcitabine [107,117], although its use may result in damage to the fetus, 2′-2′-C-methylcitosine, 2′-C-methyluridine [118], and their proTides [118], 5-fluorouracil, an anticancer drug, 6-azauridine, an antineoplastic, and finasteride, used in patients with enlarged prostate [97,108,119], also reduce virus multiplication. However, the clinical use of these last two may be impaired by their low solubility. Likewise, P12-23 and P12-34 derivatives of AR-12, an anticancer agent, suppress ZIKV replication in vitro and significantly improves the survival of infected mice [120]. Sofosbuvir, approved by the FDA to treat HCV infected patients, inhibits ZIKV replication in human cell lines of different origin origin [121,122] and improves survival rates in infected susceptible mice, origin [121], though it is not recommended for use in pregnant women. Viperin, an interferon-inducible protein, catalyzes the conversion of cytidine triphosphate (CTP) to 3’-deoxy-3’,4’-didehydro-CTP (ddhCTP), which acts as a chain terminator for the RNA-dependent RNA polymerase and, thus, inhibits replication of Zika virus in mice [123,124]. Aurintricarboxylic acid (ATA), a polyanionic aromatic compound and a potent ribonuclease inhibitor with apoptotic activity that inhibits several bacteria and viruses in vitro, has also been shown to reduce ZIKV multiplication in pre- and post-infection settings [125]. Finally, the methyltransferase domain of the NS5 protein that is responsible for transferring the mRNA cap to the 5’ end of the viral genomic RNA, and which structure is already known, may also serve as a target for the design of antivirals against ZIKV, as it has been demonstrated by the inhibitory activity of several compounds, F3043-0013, F0922-0796, F1609-0442, and F1750-0048 [126,127,128].

#### 3.1.3. Protease Inhibitors. 

The N2B-NS3 trypsin-like serine-protease and the NS3 helicase contribute to viral polyprotein processing and, thus, have an important role on virus replication. As a result, several reports have searched for antiviral against these viral enzymes [146,147,148]. 

An early report identified seven natural flavonoids that inhibited ZIKV multiplication [129]. Additional natural flavonoids like myricitin, quercetin, luteolin, isorhamnetin, and apigerin, and the phenol curcumin inhibit the N2B-NS3 noncompetitively [130], although some of them seem to be promiscuous drugs, as it has been reported that they affect viral binding [130,131,132]. A similar case is suramin, an anti-parasitic, that inhibits NS2B/NS3 complex [133], but also viral binding to the cell membrane [131,132]. On the other hand, by screening a battery of almost 3000 approved and investigational drugs, three compounds (temoporfin, niclosamide, and nitazoxanide) were identified that presented inhibitory activity against ZIKV and also DENV, JEV, YFV, and WNV [134]. Even more, temoprofin inhibited viremia and protected mice against lethal infection [134] In this line, by using a structure-based assay over 8000 approved and investigational drugs, various potent non-competitive NS2B-NS3 inhibitors were identified [135], among them, novobiocin, which can be administered to pregnant women, significantly increases survival rates of infected mice, and reduces blood and tissue viral levels, as well as histopathological damages [135]. By molecular docking studies it was predicted that bromocriptine bind to the NS2B-NS3, inhibiting its proteolytic activity, which was further confirmed in cell culture assays [136]. An additional study tested several HCV NS3-NS4A protease inhibitors and found that two inhibited ZIKV NS2B-NS3 probably binding to the protein active site [137]. Later on, it was reported that aprotinin, a bovine trypsin inhibitor with activity against WNV, also reduces ZIKV multiplication [138]. However, although molecular modeling predicted that it probably blocks the NS2B/NS3 interaction, others study with WNV and DENV indicated that it binds and occludes the enzyme substrate site [139,140]. In this line, another study screened hundreds of protease inhibitors and found three (NSC157058, NSC86314, and NSC716903) that inhibited the enzyme [138]. In fact, NSC157058 significantly decreased viremia in infected mice, but it had an unfavorable pharmacokinetic profile [138]. Another DENV inhibitor, NSC135618, was shown to also inhibit ZIKV as well as WNV and YFV, although the mechanism has not yet been elucidated [141]. Later on, it has been reported that erythrosin B, an FDA-approved food additive, inhibits the ZIKV and DENV2 NS2B-NS3 proteases by a non-competitive mechanism [142]. Finally, viperin was found to interact and colocalize with the ZIKV nonstructural proteins NS2A, NS2B, and NS3, reducing NS3 expression by induction of its proteasome-dependent degradation, inhibiting viral multiplication, an effect that was not observed for JEV or YFV [123], although it has also been reported that acts as pyrimidine inhibitor [124].

On the other hand, searching for inhibitors of the NS3 helicase and the NS4B presents several drawbacks that are complicating its development, although preliminary studies with DENV may open a door to find compounds acting against these ZIKV proteins [149].

### 3.2. Host-Acting Antivirals (HAAs)

ZIKV, as a flavivirus, has a small RNA genome (~ 10.7 Kb in length), which implies that it requires different host factors and the use of cellular metabolic pathways to propagate efficiently [150]. Thus, this may be used in search of host targets as therapeutic tools. Even more, since different members of the family *Flaviviridae* often share these host factors, these compounds have the potential of being pan-flaviviral antivirals, and its use would be less prone to the emergence of escape mutants, as often occurs with the DAAs [151,152]. Even though interfering with host factors and metabolism has raised concerns, drugs as ibuprofen and aspirin (COX-2 ciclooxigenase inhibitors) or statins (3-hydroxy-3-methyl-glutaryl-coenzyme A, HMG-CoA reductase inhibitors) are nowadays widely used in the clinical practice. Therefore, HAAs interfering with almost all steps of ZIKV life cycle (binding, entry, fusion, translation, transcription, replication, maturation, and egress) have been evaluated (Table 3). 

#### 3.2.1. Early Steps

As in any viral infection, the initial step is the binding of ZIKV to the receptor anchored in the cell membrane and its internalization into the cytoplasm. So far, several molecules have been suggested to be ZIKV receptors (AXL, DC-SIGN, Tyro3, TIM and TAM). These receptors are expressed in different permissive cell types, and, in some instances, abrogation of a given receptor can drive to the use of a different one [153,210]. Inhibition of viral binding has been achieved in different cell types with various molecules, such as R428 and cabozantinib (AXL kinase inhibitors) [153,211], MYD1 (an AXL decoy) [154], curcumin (a food additive) [155], and suramin (an anti-parasitic) [131,132], although it has also been described that it inhibits the NS2B/NS3 complex [133]. Similarly, nonsteroidal anti-inflammatory drugs (NSAIDs), such as ibuprofen, aspirin, acetaminophen, lornoxicam, and naproxen have been reported to prevent the entry of the Zika virus, in some instances by reducing the expression of AXL, the entry cofactor of ZIKV; [156] however, NSAIDs are usually contraindicated as in other flavivirus infection they increase the risk of the hemorrhagic syndrome [212]. On the other hand, since lipids are essential components of ZIKV virions, it has been described that the small molecule CLR01 [157] and an amphipathic α-helical peptide [158] both disrupt virion envelope, reducing ZIKV-infectivity in cell cultures. Even more, the latter showed therapeutic effect on infected mice.

After binding, ZIKV is internalized by endocytosis to reach the endosomes [154] where the viral genome is released into the cytoplasm after endosomal acidic pH triggers the fusion of the viral envelope with endosomal membranes [213], a process dependent of the presence of cholesterol and specific lipids in the membrane [214] These processes have been inhibited by different compounds such as nanchangmycin, an insecticide and antibacterial [107], the broad-spectrum antivirals arbidol [159] and compound 16, a pyridobenzothiazolone that does not reduce ZIKV RNA synthesis, but prevents a second round of infection [160], chlorpromazine, an antipsychotic [161], and daptomycin, an antibiotic [104].

Obatoclax (or GX15-070), an inhibitor of Bcl-2, reduces endosomal pH and inhibits ZIKV fusion to the membranes [117,162], but because of low solubility it was not effective when tested for hematological and myeloid diseases in clinical trials. Saliphenylhalamide (SaliPhe) also blocks endosomes acidification and inhibits ZIKV multiplication in virus natural human cells targets [108,117] .Likewise, amodiaquine, an antimalarial drug [163], inhibits ZIKV mutiplication in cell culture, as does the scorpion *Euscorpiops validus* venom peptide Ev37 that alkalizes acidic organelles preventing low pH-dependent fusion of the viral membrane-endosomal membrane [164]. Chloroquine (CQ), a FDA-approved anti-inflammatory widely used as an anti-malarian drug, inhibits the fusion to the endosomal membrane by raising the endosomal pH [166,168]. CQ also reduces placental and fetal ZIKV infection [167], and attenuates ZIKV-associated morbidity and mortality, protecting mice foetus from microcephaly [165], and vertical transmission in ZIKV-infected pregnant mice, significantly reducing foetal brain viral loads [138]. Other lysosomotropic agents (ammonium chloride, mefloquine, quinacrine, bafilomycin A1, and GSK369796) that neutralize the endosomal acidic pH also block ZIKV infection of different cell types [161,168]. Similarly, different molecules that interfere with the endosomal activity, like the small inhibitor K22 that produces severe alterations of ZIKV-induced intracellular replication compartments [169], and iron salt ferric ammonium citrate (FAC) [170], tenovin 1 [107], and niclosamide [171], a category B antihelmintic drug approved by FDA, also inhibit ZIKV multiplication. Niclosamide also decreases ZIKV production and prevents apoptosis in human cells, and partially rescues ZIKV-induced microcephaly and attenuates infection in a developed humanized ZIKV-infected embryo model in vivo [172]. Overall, all these molecules have demonstrated its potential for targeting ZIKV entry and internalization in cell culture.

#### 3.2.2. Transcription/Translation

Once ZIKV-RNA is in the cytoplasm, a negative strand RNA is synthesized to direct the production of the new positive strand RNA molecules for their further encapsidation [4]. Different compounds targeting these processes have been assayed as ZIKV antivirals. Silvestrol, a natural compound that inhibits eIF4A, and fenretinide (or 4-HPR), an inhibitor of cancer proliferation that induces apoptosis, limit ZIKV replication in human cell lines [173,174], and the later one significantly reduces viremia and brain viral burden in mice [174]. Likewise, drugs interfering with the polyamine biosynthetic pathway needed for ZIKV translation and transcription [215], as difluoromethylornithine (DFMO or eflornithine), an FDA-approved drug to treat trypanosomiasis and some cancers, and diethylnorspermine (DENSpm) impair viral multiplication [216]. On the other hand, the small molecule GW5074 inhibits the interaction between the NS5 and the host importin IMP α/β1 and, thus, NS5 nuclear localisation [175].

#### 3.2.3. Replication

ZIKV replication and particle morphogenesis are carried out in the ER and then, once packaged, inmature virions traffic thru the trans-Golgi before cleavage of the prM protein to render mature virions that are released from the cell [4]. Thereby, different drugs that affect these processes have been shown to inhibit ZIKV multiplication. Brefeldin A [176], a *Penicillium sp* inhibitor of protein transport from the ER to the Golgi, emetine [177], an anti-protozoal that disrupts lysosomal function, NGI-1 [178], a modulator of the oligosaccharyltransferase (OST) complex, the host ER-associated signal peptidase (SPase) [179], cavinafungin [180], a fungal compound, and nitazoxanide [181], an FDA approved broad-spectrum antiviral, all impair ZIKV infection in different cell types.

Cell death during ZIKV infection is mediated by the induction of host caspases-3 and neuronal apoptosis [217]. Therefore, caspases inhibitors as bithionol [182], and emricasan [171], currently in phase 2 clinical trials for chronic HCV, inhibit ZIKV in cells of different origin. Likewise, phloretin reduces ZIKV infection by decreasing apoptotic caspase-3 and -7 activities and by reducing AKT/mTOR phosphorylation pathways, which, together with the fact that 2-deoxy-D-glucose disruption of cellular glucose availability inhibits ZIKV propagation, remarks the importance of glucose pathways for ZIKV propagation [183]. Additionally, Bortezomib [104], a proteasome inhibitor approved for treatment myeloma and non-Hodgkin’s lymphoma, and PHA-690509 [171], a cyclin-dependent kinase (CDK) inhibitor, also reduce ZIKV-infection and propagation, although the latter is not adequate for use during pregnancy.

Host lipids are essential for flavivirus replication and particle formation and, thus, they are potential candidates for antiviral intervention [214,218]. Accordingly, two drugs that interfere the sterol regulatory element-binding proteins (SREBP) pathway and disturb the lipid metabolism, nordihydroguaiaretic acid (NDGA) and its derivative the tetra-*O*-methyl nordihydroguaiaretic acid (M4N), which is currently in Phase I/II clinical trials in patients with advanced cancer, inhibit the infection of ZIKV, as do other structurally unrelated inhibitors of the SREBP pathway, such as PF-429242 and fatostatin [184]. Similarly, three acetyl CoA carboxylase (ACC) inhibitors (PF-05175157, PF-05206574, and PF-06256254) also impair ZIKV replication in cellular culture [185] In addition, activation of adenosine monophosphate-activated protein kinase (AMPK), a regulator of lipid metabolism and ACC, by PF-06409577, metformin, and 5-aminoimidazole-4-carboxamide ribonucleotide (AICAR) were shown to reduce ZIKV replication [186,187].

Cholesterol is also a key player during flavivirus infection. So that, the cholesterol derivative 25-hydroxycholesterol (25-HC) [188,189] reduces mortality and prevents microcephaly in ZIKV-infected mice, and decreases viral loads in the urine and serum of NHP [189]. Likewise, 7-ketocholesterol (7-KC) that probably acts by inducing cellular autophagy, also reduces ZIKV replication [190], as do imipramine, an FDA-approved antidepressant, and benzamil that interfere with intracellular cholesterol transport [191,192]. Two other HMG-CoA inhibitors widely used to control cholesterol in the clinical practice, lovastatin, which attenuates nervous injury in animal model of Guillain–Barré syndrome, Sarkey [193], and mevastatin have been proposed as ZIKV antivirals [120]. In addition, it has been shown that the neutral sphingomyelinase inhibitor GW4869 reduces ZIKV production by affecting viral morphogenesis [194].

#### 3.2.4. Other Compounds Acting Against ZIKV Infection

In addition to the above mentioned antivirals, some other drugs have been reported as effective against ZIKV infection in cell culture, although the specific mechanism by which they act is currently unknown. In this way, the antibiotics kitasamycin [120], a natural product from *Streptomyces narbonensis*, and azithromycin [195] prevent ZIKV infection in cell culture, as do cyclosporine [104], an immune depressor currently in clinical trials for its possible use in ameliorate neuronal cellular damage, and the antidepressant sertraline [120], a selective serotonin reuptake inhibitor. Similarly, the antiparasitics ivermectin, used to treat worms infections, pyrimethamine, used for toxoplasmosis and cystoisosporiasis [104], and amodiaquine dihydrochloride dihydrate (AQ) [196], an FDA approved drug for treatment of malaria, also inhibit ZIKV infection of human cells, and the last one also in adult mouse brain even after infection has progressed. Palonosetron, a FDA approved drug to prevent chemotherapy-induced nausea and vomiting, has also been proposed for treatment of ZIKV infection [120]. In addition, Hsp70 isoforms are recruited for ZIKV entry, replication, and assembly, and, thus, compounds targeting them reduce viral multiplication in several cell lines, and protect mice, reducing viremia, mortality, and disease symptoms [197].

On top of HDAs, some compounds have been shown to be effective in preventing side effects linked to ZIKV infection. So that, blockers of the N-methyl-D-aspartate receptor (NMDAR), implicated in neuronal damage that often occurs during ZIKV infection [198,219,220], such as ifenprodil, dizocilpine agmatine sulfate, and memantine, used to treat patients with Alzheimer’s disease, prevent neuronal damage in infected mice, but do not inhibit viral replication [199]. Ebselen (EBS), an antioxidant, improves testicular injury in mice by reducing oxidative stress, leucocyte infiltration, and production of pro-inflammatory response, and also prevents virus sexual transmission, but it neither reduces viremia nor improves survival rate [200]. IL-1 receptor antagonist (IRA; Kineret, or anakinra) can preserve placental, increase fetal viability, attenuate fetal neuroinflammation, behavioral deficits, and improve perinatal outcomes [201].

#### 3.2.5. Modulators of the Innate Immunity

ZIKV infection, as many other viruses, activates the innate immune response by activation of interferons (IFNs) stimulated genes to confer resistance to viral infections [16,221,222]. Therefore, IFNs inhibit ZIKV replication in cell culture [202,203] and in mice [204], as did IFN-induced transmembrane proteins (IFITM1 and IFITM3) [205], interferon-activating small molecule AVC [206], and the scorpion venom peptide (Smp76) from *Scorpio maurus palmatus* that suppresses ongoing viral infection by upregulating the expression of IFN-β activating interferon regulatory transcription factor 3 (IRF3) phosphorylation [207]. Additionally, several small human noncoding microRNAs (miRNAs) that affect gene expression and regulate many different cellular processes have been shown to inhibit ZIKV by activating the IFN-based innate immune response [208]. Nonetheless, ZIKV can also evade type I IFN response, [223,224,225,226] which may lead to spontaneous abortions and growth restriction during pregnancy [227]. Finally, it has been described that inhibitors of epigenetics modulators such as GSK-126 that inhibits histone H3K27 methyltransferases, a suppressor of gene transcriptions, reduces ZIKV multiplication in cell culture by activating the cellular antiviral and immune responses [209].

### 3.3. Future Perspectives for ZIKV Antivirals.

As in the case of vaccine development, a great effort is being made to find compounds to fight ZIKV infection by applying different approaches, from the screening of bioactive molecules from different libraries to repurposing drugs with known antiviral activity and the use of natural products. However, it should be noted that many different methodologies, viral strains and cell types have been used, which sometimes has driven to contradictory results with the same compound. On top of that, most of the antivirals candidates have been tested in vitro, and only a few have been assayed in vivo, which hampers the interpretation of the results, as extrapolation of in vitro results to in vivo is usually difficult. In fact, in the occasions in which in vivo experiments have been conducted in animal models they have often failed to give the expected outcomes, and thus, this points to the complication for most of them to complete the entire drug development pipeline. In addition, as many of the tested candidates may have untoward effects, careful evaluation should be conducted before they can enter in the clinical practice, mainly when the main target population will be pregnant women and patients with other medical complications.

## 4. Conclusions

In as little as three years, a great number of vaccine and antiviral candidates has been tested in cell culture and animal models. Most of the assayed vaccines have produced hopeful results (potent specific humoral and cellular responses, decrease of viremia and viral RNA in natural target organs, prevention of vertical viral transmission, development of microcephaly, and protection in experimental animal models), and even some of them have entered in clinical trials. However, several questions remain to be deeply analyzed and resolved before any of these candidates can be implemented in the field. For instance, as other flaviviruses co-circulate with ZIKV in the same regions, avoiding adverse effects such as exacerbation of disease due to, for example, the induction of ADE is required. Likewise, and based on previous experiences with other flaviviruses vaccines, safety for use in pregnant women is mandatory. Even more, vaccines should not only protect from the ZIKV infection, but ideally also prevents replication in sexual organs and viral spread. The cost-effectivity of vaccine campaign implementation should also be considered. Similarly, and even though dozens of antiviral candidates (DAAs and HAAs) have been tested that affect different aspects of the viral life cycle, few of them have been assayed in animal models, and safety and untoward effects should be carefully evaluated before they can be used in clinical practice. Therefore, a long way is still ahead before therapeutic measures against ZIKV can be applied. However, the public health need for ZIKV vaccines and antivirals persists. Although the number of human cases have dropped a lot since 2017, investigations should go on, since, as flaviviruses share many biological characteristics, their implementation should not only be pertinent for ZIKV infections, but also for that of other currently circulating flaviviruses, and for those possible yet unrecognized, or almost neglected, flaviviruses that can emerge in the future.

## Figures and Tables

**Table 1:** Zika virus (ZIKV) vaccines clinical trials.

Platform	Vaccine	Antigen	Adjuvant	Regimen (Dose, Route, Day/Week)	Clinical Phase	Trial Number	References
Inactivated	BBV121	virion	Alum	2.5 vs. 5 vs. 10 mg 2× (0, + 30)	I	CTRI/2017/05/008539	[20,21]
	ZPIV	virion	Alum	5 mg/IM 2× (+ 1, + 29)	I	NCT02963909	[22,23]
	ZPIV	virion	Alum	2.5 vs. 5 vs. 10 mg/IM 2× (0, + 29)	I	NCT02952833	[22,23]
	ZPIV	virion	Alum	5 mg/IM 2× (0, + 7: 0, + 14; 0, + 29)	I	NCT02937233	[22,23]
	ZPIV	virion	Alum	YF-VAX/IXIARO-5 mg/IM 2× (+ 1, + 29)	I	NCT03008122	[22,23]
	PIZV	virion	Alum	2 vs. 5 vs. 10 mg/IM 2× (0, + 30)	I	NCT03343626	[22,23]
	VLA1601	virion	Alum	3 vs. 6 AgU 2× (0, + 1 vs. 0, + 4)	I	NCT03425149	[22,23]
DNA-based	GLS-5700	pM/E	None	1 mg/ID	I	NCT02809443	[24,25,26]
	GLS-5700	pM/E	None	2 mg/ID	I	NCT02887482	[24,25,26]
	VRC5283	pM/E	None	4 mg/IM both arms, 2× (0, + 8; 0, + 12) 3× (0, + 4, + 8; 0, + 4, + 20)	I	NCT02840487	[27,28]
	VRC5283	pM/E	None	4 mg/IM both arms, 2× (0, + 8) (0, + 12); 3× (0, + 4, + 8) (0, + 4, + 20), needle/needle-free	I	NCT02996461	[27,28]
	VRC5283	pM/E	None	4 mg vs. 8 mg/IM both arms, 3× (0, + 4, + 8) needle-free	II	NCT03110770	[27,28]
RNA-based	mRNA-1325	pM/E	None	mRNA-1325	I	NCT03014089	[29,30]
Recombinant	MV	pM/E	None	low/high dose (0 vs. 0, + 30)	I	NCT02996890	[31]

**Table 2:** Direct acting antivirals (DAAs) against ZIKV.

Step	Proposed Target	Compounds	System Used to Test the Drugs	References
Binding	Preventing E binding	Neutralizing antibodies	Cell culture, biochemical assays, and animal models	[68,69,70,71,72,73,74,79]
	Virion morphology and E-mediated membrane fusion & entry	Z2, Porphyrins, BZM-CDs, POMs, SCRs, small molecules,		[80,81,82,83,84,85,86,87,88,89]
	Viricidal	Croton, *Aphloia theiformis* and *Psiloxylon mauritianum* extracts, epigallocatechin gallate, delphinidin, pinocembrin		[90,91,92,93,94,95,96]
Replication	NS5 polymerase inhibitors (Purine synthesis inhibitors)	Ribavirin, merimepodib, favipiravir, mycophenolic acid, azathioprine, methotrexate, avotaquone, 6-methylmercaptopurine riboside, atovaquone, 2’-CMG, 2’-CMA 7-deaza-2’-CMA, NITD008, BCX4430	Cell culture, biochemical assays, and animal models	[97,98,99,100,101,102,103,104,105,106,107,108,109,110,111,112,113,114,115]
	NS5 polymerase inhibitors (Pyrimidine synthesis inhibitors)	Brequinar, CID91632869, gemcitabine, 2’-CMC, 2’-CMU, 5’-fluorouracil, 6-azauridine, finastenide, P12-23 and P12-34, sofosbuvir, viperin, aurintricarboxylic acid	Cell culture, biochemical assays, and animal models	[97,107,108,110,116,117,118,119,120,121,122,123,124,125]
	NS5 methyltransferase inhibitors	F3043-0013, F0922-0796, F1609-0442, and F1750-0048, Compound 10	Biochemical assay, docking	[126,127,128]
	Viral protease inhibitors	myricitin, quercetin, luteolin, isorhamnetin, apigerin, compound 2, compound 3, curcumin, suramin, temoporfin, niclosamide, nitazoxanide, novobiocin, bromocriptine, aprotinin, NSC157058, NSC86314, NSC716903, NSC135618, erythrosin B, viperin	Cell culture, biochemical assays, and animal models	[129,130,131,132,133,134,135,136,137,138,139,140,141,142]

**Table 3:** Host acting antivirals (HAAs) against ZIKV.

Step	Proposed Target	Compounds	System Used to Test the Drugs	References
Early Steps	Receptor binding inhibition	R448, cabozantinib, MYD1 curcumin, suramin, NSAIDs (ibuprofen, aspirin, acetaminophen, lornoxicam, naproxen), CLR01, amphipathic α-helical peptide	Cell culture	[131,132,153,154,155,156,157,158]
	Internalizationfusion inhibitors/endosome acidifcation	Nanchangmycin, arbidol, compound 16, chlorpromazine, daptomycin, Obatoclax, SaliPhe, amodaquine, peptide Ev7, CQ, ammonium chloride, mefloquine, quinacrine, bafilomycin A1, and GSK369796, K22, iron salt ferric ammonium citrate, tenovin 1, niclosamide	Cell culture, human organoids, animal models	[104,107,108,117,138,159,160,161,162,163,164,165,166,167,168,169,170,171,172]
Translation/Transcription		Silvestrol, fenretinide, DFMO, DENSpm, GW5074	Cell culture	[155,173,174,175]
Replication	Intracellular transport	Brefeldin A, emetine, NG-1, SPase, cavinafungin, nitazoxanide	Cell culture	[176,177,178,179,180,181]
	Caspases/CDK inhibitors	Bithionol, emricasan, phloretin, bortezomib, PHA-690509	Cell culture	[104,107,171,182,183]
	Lipids metabolism	NDGA, M4N, PF-429242, fatostatin, metformin, AICAR, PF-05175157, PF-05206574, PF-06256254, PF-06409577	Cell culture	[184,185,186,187]
	Cholesterol metabolism	25-HC, 7-KC, Imipramine, benzamil, lovastatin, mevastatin, GSW4869,	Cell culture, animal models	[120,188,189,190,191,192,193,194]
Unknown		Kitasamycin, azithromycin, cyclosporine, sertraline, ivermectin, pyrimethamine, AQ, palonosetron, Hsp70-NEF interaction inhibitors	Cell culture, animal models	[104,120,195,196,197]
Side effects		ifenprodil, dizocilpine agmatine sulfate, memantine, ebelsen, IRA	Cell culture, animal models	[198,199,200,201]
Innate immunity modulation		IFNs, IFITM1 and IFITM3, AVC, Smp76, miRNAs	Cell culture, animal models	[202,203,204,205,206,207,208]
Epigenetics		GSK-126	Cell culture, animal models	[209]
